# Characterization of microsatellite markers in the coding regions of the *Penaeus vannamei* genome

**DOI:** 10.1371/journal.pone.0289351

**Published:** 2024-05-02

**Authors:** Iasmim Santos Mangabeira-Silva, Paulo Eduardo Toscano Soares, Yago Tomaz Vieira da Silva, Beatriz Helena Dantas Rodrigues de Albuquerque, Maryana Thalyta Ferreira Câmera de Oliveira, Larissa Alves Honorato Ferreira, Maria Fernanda Bezerra de Souza, Danyllo Vieira de Lucena, Jessica Marina Paiva Pereira, Roseli Pimentel Pinheiro e Silva, Daniel Carlos Ferreira Lanza

**Affiliations:** 1 Laboratory of Applied Molecular Biology—LAPLIC, Federal University of Rio Grande do Norte, Natal, RN, Brazil; 2 Postgraduate Program in Biotechnology, RENORBIO, Natal, RN, Brazil; 3 Postgraduate Program in Biochemistry, Federal University of Rio Grande do Norte, Natal, RN, Brazil; 4 Postgraduate Program in Civil and Environmental Engineering, Federal University of Campina Grande, Campina Grande, PB, Brazil; 5 Samaria Unidade de Pós Larvas, Touros, RN, Brazil; National Cheng Kung University, TAIWAN

## Abstract

In this study, an extensive analysis of microsatellite markers (Single Tandem Repeats—STRs) in *Penaeus vannamei* was conducted at an advanced level. The markers were thoroughly examined, characterized, and specific markers located within coding regions were identified. Out of a total of 306 STRs, 117 were classified as perfect markers based on their single repeat motif. Among these perfect markers, 62 were found to be associated with predicted coding genes (mRNA), which were involved in various functions such as binding, catalytic activity, ATP-dependent activity, transcription, structural and molecular regulation. To validate the accuracy of the findings, a sample of nine markers was subjected to *in vitro* testing, which confirmed the presence of polymorphisms within the population. These results suggest the existence of different protein isoforms within the population, indicating the potential of these markers for application in both population and phenotype-genotype association studies. This innovative approach opens up new possibilities for investigating the impact of genomic plasticity in populations of *P*. *vannamei*.

## Introduction

An inherent characteristic of marine shrimp production systems is the increase in inbreeding within populations, which leads to a higher genetic similarity among individuals. Consequently, this can result in undesired production traits, such as increased susceptibility to pathogens [1–3]. Genetic selection plays a crucial role in identifying desirable phenotypes and maintaining genetic diversity, both of which are essential for successful shrimp farming [4, 5].

In this context, the study and identification of microsatellite markers, also known as Short Tandem Repeats (STRs), hold great potential for practical applications. These markers offer a cost-effective means to monitor individual genetic characteristics [6, 7]. Microsatellites possess several advantageous features, including broad genome coverage, multi-allelic nature, co-dominance, and polymorphism, making them excellent tools for studying genetic variation and constructing genetic maps [8, 9].

Among the STR markers, those composed of simple sequences (e.g., consisting of a single repeated motif like ACACAC…AC, or simply ACn) are referred to as perfect markers. These markers are preferred for panel assembly due to their high accuracy in genotyping breeding populations. Their repetitive and straightforward structure facilitates efficient genotyping, making them an excellent choice in breeding programs. These markers can be categorized into mono-, di-, tri-, tetra-, penta-, and hexanucleotides, based on the length of their repeat motif [10]. The variation in the number of motif repeats often provides insights into the possible alleles present at each locus, making it easier to determine bin sets. This information is crucial for automating the genotyping process [11–13].

During their early development, STRs were primarily regarded as neutral markers [14, 15]. However, in recent years, numerous studies have demonstrated associations between these markers and known genes or specific phenotypes. This growing body of research supports the notion that certain STRs play an evolutionary role by serving as significant sources of adaptive genetic variation [14–17].

The association of STR markers with specific phenotypes in marine shrimp *Penaeus vannamei* has been relatively unexplored, primarily due to the availability of new SNP-based genotyping methods [18]. Unlike SNPs, which typically require around 60 to 100 markers for genotyping individuals, a small number of STRs (sometimes even fewer) can accomplish the same task [19, 20]. This difference opens up a range of possibilities for discoveries, considering that approximately 24% of the *P*. *vannamei* genome is composed of STRs, and within this fraction, around 78% are found in genes that potentially encode proteins [17, 21].

Therefore, the objective of this study was to further investigate and characterize all previously described microsatellite markers in *P*. *vannamei*. Specifically, the focus was on the functional characterization of perfect markers located within coding regions. Additionally, a multiplex PCR panel using a small set of perfect STRs was proposed. The results obtained in this study emphasize the significance of STRs within protein-coding regions and contribute to the development of a new STR-based multiplex genotyping panel for *P*. *vannamei*. This panel offers a cost-effective alternative for genetic selection programs, providing valuable tools for breeders in their efforts to improve the species.

## Material and methods

### Microsatellite search on *Penaeus vannamei*

Literature on STRs in *P*. *vannamei* was obtained from the NCBI (https://www.ncbi.nlm.nih.gov/) using the following ENTREZ query: "*microsatellite repeats"[MeSH Terms] OR ("microsatellite"[All Fields] AND "repeats"[All Fields]) OR "microsatellite repeats"[All Fields] OR "microsatellites"[All Fields]) AND shrimp[All Fields] AND ("penaeidae"[MeSH Terms] OR "penaeidae"[All Fields] OR "penaeus"[All Fields]) AND vannamei[All Fields]*.

Based on this investigation, a comprehensive database of STRs was established. These markers were categorized based on their type, whether they were perfect or composite, as well as their specific motif and number of repeats. The database also included information about the position of each marker within the genomic sequence, the primer sequences used for amplification (both forward and reverse), the melting temperature (TM) of each primer, the number of alleles detected for each STR in the tested populations, and the name of the corresponding author.

For subsequent analyses, only the markers classified as perfect STRs were chosen and utilized.

### Identification of microsatellites within coding sequences

The *in silico* identification of the sequences flanking each marker (i.e.: the amplicon sequence) was performed using Primer-BLAST (https://www.ncbi.nlm.nih.gov/tools/primer-blast/). The forward and reverse primers for each STR were used as the search input, the ‘database’ was set to “Refseq representative genomes” and the “organism” parameter was set to “*Penaeus vannamei* (taxid:6689)”. The parameter “max target amplicon size” was adjusted to 500 bp based on the average size of the alleles previously described.

Sequences in which the primers showed a 100% identity match or had at most 1 base pair difference (SNP or deletion) were considered for further analysis. The flanking regions, including the primers, also known as putative amplicons, were manually extracted. This process involved using the provided coordinates of the aligned primers and ensuring that the entire amplicon region was captured, starting from the left-most coordinate, and ending at the right-most coordinate in the corresponding sequence. In cases where confirmation of microsatellites was necessary, the Geneious software version 7.1.3 [22] was employed.

Subsequently, the sequences of the putative amplicons were functionally annotated using BLASTx [23] against the ’nr’ database, employing default parameters. Protein functions were assigned to the sequences based on their best alignment (best hit). This analysis aimed to identify putative genes encoded by the sequences aligning with each marker. By associating the marker sequences with known protein functions, we gained insights into the potential genetic elements represented by the markers.

Next, we determine genomic locations of these marker sequences in the *P*. *vannamei* genome, unveiling the associated genomic features based on the provided genome annotations [21].

For this analysis, we exclusively selected markers that yielded a single hit during the Primer-BLAST step to ensure accuracy and eliminate potential ambiguities caused by multiple targets within the same or different genomic scaffolds. In essence, we conducted a BLASTn search to obtain the genomic location of each marker, employing a ’best hit’ approach. Subsequently, we examined the genome annotation file (GFF file) directly using the BEDTools software v2.26.0 to identify any overlapping annotations or features associated with the markers [24]. First, BLASTn from the NCBI BLAST+ package v2.9.0 [25] was run locally (parameters: -task blastn -outfmt 6) to align the marker sequences to the *P*. *vannamei* genome, assigning each marker’s location to the one in its best hit.

A second BLASTn analysis was conducted using the Lvan99 sequence, which exhibited 90% low complexity and initially failed to align. To overcome this issue, the second BLASTn search was performed with the same parameters as before but with the DUST filter deactivated (-dust no), resulting in successful alignments. The results from both BLASTn analyses were then merged.

For each aligned marker sequence, the corresponding subject ID (representing the scaffold ID), start and end positions of the alignment in the subject, and query ID (representing the marker ID) were extracted. These details were formatted into a BED format consisting of four columns. The BEDtools program was utilized to search for features based on this BED file, allowing for further analysis of the aligned marker sequences in relation to genomic features [24].

The association of markers to linkage groups was performed using the information provided in the *P*. *vannamei* genome [21]. With the obtained BLAST best hits, we utilized the accession IDs of the subjects to retrieve the corresponding scaffold IDs. Subsequently, we mapped these scaffold IDs to their respective linkage groups, enabling the assignment of markers to specific linkage groups within the genome.

The BEDtools software was employed using the ’intersect’ function, following the pseudocommand with the specified parameters: $ bedtools intersect -a <Intervals>.bed -b <GenomicAnnotations.gff> -wo. This allowed the identification of all genomic annotations within the *P*. *vannamei* genome that overlapped with the genomic regions where the markers aligned. Detailed information regarding these genomic annotations was extracted from the genomic feature file (GFF file) associated with the *P*. *vannamei* genome. The results obtained from the ’BEDtools intersect’ analysis were subsequently analyzed using Python scripts, utilizing data analysis packages such as Pandas and Jupyter.

The protein identified through functional annotation using BLASTn from BEDtools was further subjected to a search in InterPro (https://www.ebi.ac.uk/interpro/). This allowed for the retrieval of Gene Ontology (GO) terms associated with the protein, providing insights into its molecular functions.

### Selection of polymorphic STR markers, PCR and genotyping

After establishing the initial database, a series of filtering steps was applied to select the perfect markers for in vitro validation in the population of *P*. *vannamei*. The selected markers were those with the highest number of repeat motifs, the highest number of alleles, and located within coding regions.

DNA extraction was carried out using total DNA from 50 individuals of *P*. *vannamei* collected from shrimp farms in the state of Rio Grande do Norte, Brazil. Approximately 30 mg of abdominal muscle from each shrimp was utilized for DNA extraction, following the instructions provided by the Nucleospin® Tissue Macherey-Nagel kit.

The selected microsatellite markers were amplified under the specific conditions previously described for each marker. Genotyping was performed using a 6.5% polyacrylamide gel stained with silver nitrate. The electrophoresis runs were conducted in a vertical tank (Owl Separation System, Thermo Fisher) for an approximate duration of 5 hours, with a voltage of 2000 V and a power of 400 W.

Allelic and genotypic frequencies and the number of expected and observed heterozygotes were calculated using the software GenAlEx v 6.503 [26, 27]. The Genepop v 4.7 software was used to calculate inbreeding coefficient values (FIS) for all loci in the population [28], available online at the website <https://genepop.curtin.edu.au/index.html>. Sequential Bonferroni corrections were performed to test the significance of the results obtained [29].

## Results

### Perfect microsatellite markers in *P*. *vannamei*

We have identified 57 articles that describe microsatellites in *P*. *vannamei* from 1996 to 2021 (Table 1). Out of the microsatellites described, 306 have been characterized in population studies and exhibit polymorphism (S1 Table), enabling a more comprehensive investigation in this study. Among these 306 microsatellite markers, 117 (38.88%) are considered perfect markers due to their single repeat motif (S2 Table).

**Table 1:** Articles containing information about STRs in *P*. *vannamei*.

Category	Papers
Plasticity and adaptive evolution	[17]
Population genotyping	[30]
Identification of STRs by transcriptome	[31–33]
Analysis of genetic diversity	[1–3, 34–56]
Genomic sequencing	[21]
Association of STRs with viral resistance	[6, 16]
Association of STR with growth	[7]
Analysis of paternity	[57–61]
Comparison between STRs and SNPs in pedigree analysis	[20]
High density linkage map	[9, 52]
Heritability	[62]
EST–STR	[63–67]
Low density linkage map	[8, 65]
STRs database	[68]
Identification of STRs	[69–74]

**Fig 1:**
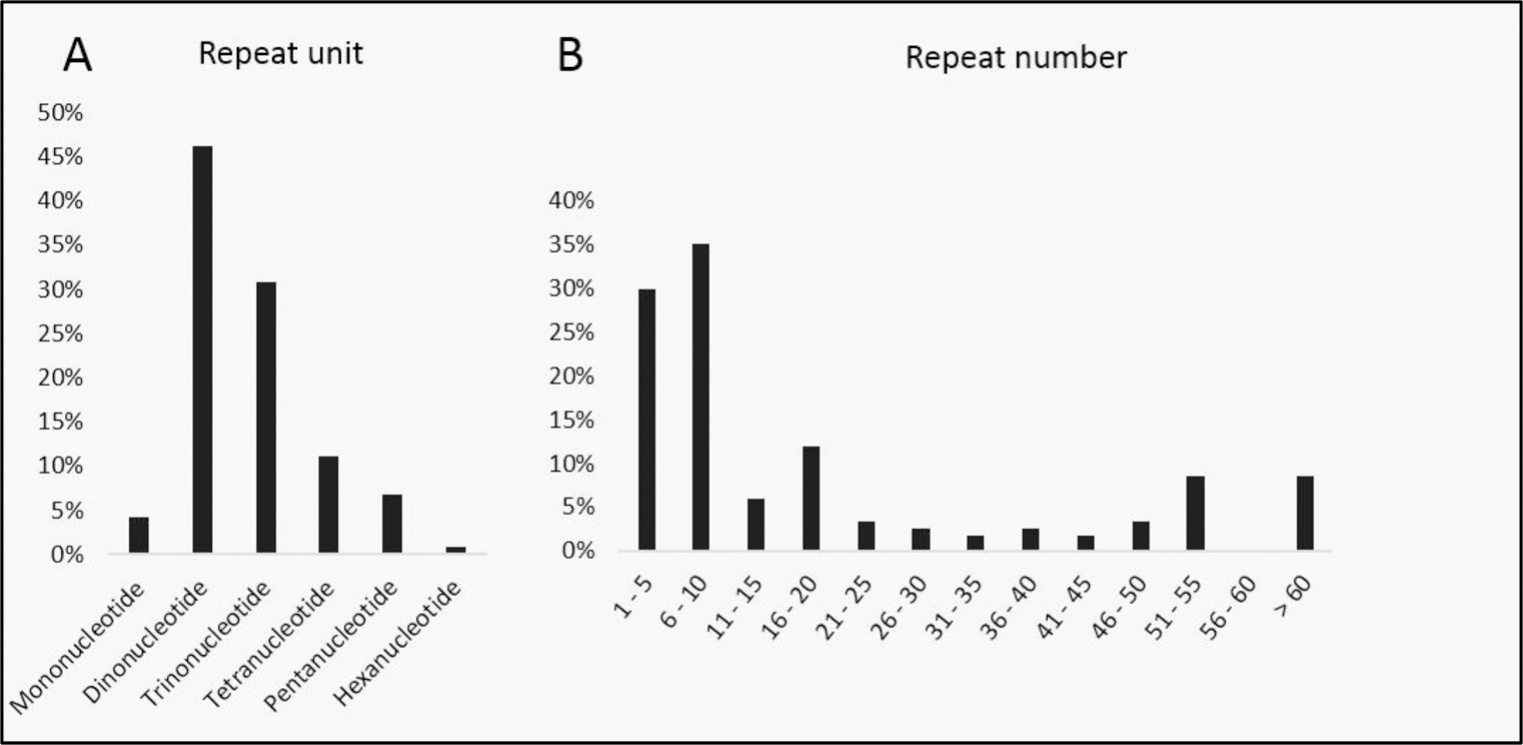
Overview and distribution of short tandem repeats (STRs). (A) Summary of di-, tri-, tetra-, penta-, and hexanucleotide microsatellites. (B) Distribution of STRs by Repeat Number.

Within the perfect markers, the highest prevalence was observed for dinucleotide (46.2%), followed by trinucleotides (30.8%) and tetranucleotides (11.1%). The mononucleotide repeats consisted of thymine repeat motifs (T), ranging from 15 to 20 repeats. One microsatellite marker exhibited a hexanucleotide repeat motif (Fig 1A). The number of uninterrupted repeats ranged from 3 to 62, with the majority consisting of short repeats. Both groups with up to 5 repetitions and the category of 6 to 10 repetitions were more frequent, comprising 34 and 43 markers, respectively (Fig 1B).

Among the perfect markers, 55 have their sequences deposited in GenBank, while 62 do not. Therefore, we identified their sequences in the *P*. *vannamei* genome using Primer-BLAST. This process resulted in obtaining 135 target sequences, as seven markers aligned in multiple regions of the genome, producing similar target sequences. These markers include CNM-MG-444 (n = 3), CNM-MG-474 (n = 2), CNM-MG-369 (n = 2), c7152/f1p0/6395_1 (n = 2), c7152/f1p0/6395_2 (n = 2) c7152/f1p0/6395_3 (n = 2) and c16138/f14p37/618 (n = 19). No target sequences were found for seven loci: Livan10, Livan13, Livan56, Lvan33(H07-2), CNM-MG-489, CNM-MG-498, and c2322/f15p12/3591, consequently, these markers were excluded from further analyses. All identified sequences are provided in S3 Table.

### Functional characterization of sequences containing microsatellites

Among the 135 sequences, 72 (53.33%) were associated with the prediction of gene features in functional annotation through BLASTn. Out of these, 62 were linked to predicted coding genes (mRNA), and 53 STRs were in exons. Furthermore, the linkage group with the highest number of STR markers was LG36 (n = 9), followed by LG20 (n = 6) and LG44 (n = 4) (Table 2 and S4 Table). These sequences were then assigned Gene Ontology (GO) annotations.

**Table 2:** Putative mRNA products from aligned marker sequences as inferred from the annotations in their corresponding sequences in *P*. *vannamei* genome.

Loci (SSR)	Associated mRNA product(s) found in *P*. *vannamei* genome	Inside an exon?	Linkage Group	E-Value
c1508/f7p10/3225	Ceramide synthase 6-like	Yes	LG19	2,92E-105
c16138/f14p37/618#11	Atherin-like	Yes	LG20	6,34E-134
c16138/f14p37/618#13	Uncharacterized LOC113822663	No	LG20	1,00E-124
c16138/f14p37/618#2	Translation initiation factor IF-2-like	Yes	LG20	1,82E-134
c16138/f14p37/618#7	Atherin-like	Yes	LG20	6,34E-134
c1696/f45p16/834	Ubiquitin-conjugating enzyme E2 D2B-like	Yes	LG19	0,00E+00
c1849/f45p19/986	Cytochrome c oxidase subunit 7A2, mitochondrial-like	Yes	LG34	6,98E-107
c2358/f24p36/1137	Gamma-interferon-inducible lysosomal thiol reductase-like	Yes	LG43	2,98E-138
c2438/f35p18/1618	26S proteasome regulatory subunit 6B-like	Yes	Not assigned	7,08E-140
c7160/f1p5/6655	Uncharacterized LOC113826785	No	LG20	7,48E-66
c7165/f1p0/7649	Ring canal kelch homolog	Yes	LG20	7,08E-140
c7201/f1p1/6803	RAB6A-GEF complex partner protein 1-like	Yes	LG36	7,1E-80
c7202/f1p4/7652	Uncharacterized LOC113817378	Yes	LG14	4,71E-129
CNM-MG-339	Uncharacterized LOC113811552	Yes	Not assigned	1,23E-76
CNM-MG-351	Uncharacterized LOC113824064	Yes	LG37	3,52E-104
CNM-MG-354	Sorting and assembly machinery component 50 homolog	Yes	LG32	7,82E-93
CNM-MG-367	Ubiquitin-conjugating enzyme E2-17 kDa	Yes	LG40	1,69E-141
CNM-MG-369#1	polyadenylate-binding protein 4-like	Yes	LG36	1,76E-121
CNM-MG-372	Ubiquitin-conjugating enzyme E2 R2-like	Yes	LG43	1,00E-124
CNM-MG-384	Hemocyte protein-glutamine gamma-glutamyltransferase-like	Yes	Not assigned	3,30E-111
CNM-MG-386	Endoplasmic reticulum lectin 1-like	Yes	LG28	4,71E-129
CNM-MG-390	Cytoplasmic aconitate hydratase-like	Yes	LG37	3,48E-124
CNM-MG-402	Prefoldin subunit 3-like	Yes	LG34	2,36E-86
CNM-MG-405	Uncharacterized LOC113802172	Yes	LG36	8,80E-152
CNM-MG-406	Uncharacterized LOC113802172	Yes	LG36	2,53E-152
CNM-MG-407	Uncharacterized LOC113830050	Yes	LG32	1,17E-143
CNM-MG-412	Uncharacterized protein F21D5.5-like	Yes	LG42	1,46E-122
CNM-MG-421	Eukaryotic peptide chain release factor subunit 1	Yes	LG44	6,25E-67
CNM-MG-437	Serine/arginine repetitive matrix protein 2-like	Yes	Not assigned	3,73E-63
CNM-MG-455	Transmembrane emp24 domain-containing protein 7-like	Yes	LG15	4,91E-142
CNM-MG-474#2	Penaeidin-3a-like	Yes	LG23	3,26E-91
CNM-MG-479	Vascular endothelial growth factor D-like	No	LG43	7,27E-45
CNM-MG-487	Titin homolog	No	LG13	4,47E-149
CNM-MG-548	Ribonuclease H1-like	Yes	LG12	2,38E-126
Livan04	Receptor-type tyrosine-protein phosphatase T-like	Yes	LG9	3,54E-77
Livan25	Dedicator of cytokinesis protein 7-like	Yes	LG39	5,91E-81
Livan44	Uncharacterized LOC113818723	Yes	LG44	7,37E-120
Livan51	Inverted formin-2-like	Yes	LG15	4,10E-83
Lvan183	Sorbitol dehydrogenase-like	Yes	LG42	1,18E-130
Lvan195	26S proteasome regulatory subunit 6B-like	Yes	Not assigned	2,57E-25
Lvan204	Serine protease inhibitor I/II-like	Yes	LG27	5,9E-88
Lvan50	60S acidic ribosomal protein P0-like	Yes	LG44	5,22E-71
Lvan51	60S acidic ribosomal protein P0-like	Yes	LG44	4,31E-78
Lvan59	Neuroparsin-A-like	Yes	LG9	1,52E-122
Lvan93	ATP-dependent Clp protease ATP-binding subunit clpX-like, mitochondrial	Yes	LG1	1,83E-54
Lvan96	Peptidyl-prolyl cis-trans isomerase sig-7-like	Yes	LG4	4,52E-136
Lvan98	Uncharacterized LOC113829845	Yes	LG29	2,41E-113
LvE13X	Uncharacterized LOC113802174	Yes	LG36	0,00E+00
LvE17X	Uncharacterized LOC113802172	Yes	LG36	0,00E+00
LvE22X	Prostaglandin G/H synthase 2-like	Yes	LG16	0,00E+00
LvE26X	Trimethyllysine dioxygenase, mitochondrial-like	Yes	LG24	0,00E+00
LvE2X	Uncharacterized LOC113803693	Yes	LG11	2,70E-161
LvE39X	Mediator of DNA damage checkpoint protein 1-like	No	LG7	1,86E-36
LvE40X	Uncharacterized LOC113802174	No	LG7	1,86E-36
LvE43X	Notch-regulated ankyrin repeat-containing protein-like	Yes	LG38	0,00E+00
LvE67	Uncharacterized LOC113802174	Yes	LG36	9,36E-148
LvE71	Uncharacterized LOC113802172	Yes	LG36	0,00E+00
LvE8X	Beta-1,4-galactosyltransferase 7-like	Yes	LG26	0,00E+00
LvIRF-5’UTR	Uncharacterized LOC113823218	Yes	LG27	2,57E-99
TUMXLv10.484	Serine/threonine-protein kinase DCLK1-like	No	LG9	8,72E-159
TUMXLv8.193	Uncharacterized LOC113810398	No	LG4	2,42E-161
TUMXLv9.174	Fanconi-associated nuclease 1-like	No	LG36	0,00E+00

The GO terms for molecular function describe the biochemical activities of genes based on various biological features associated with a given protein, including "biological process," "cellular component," and "molecular function" [75]. However, since there was insufficient information available for these proteins regarding the GO categories of "biological process" and "cellular component," they were not considered in our GO analysis.

**Fig 2:**
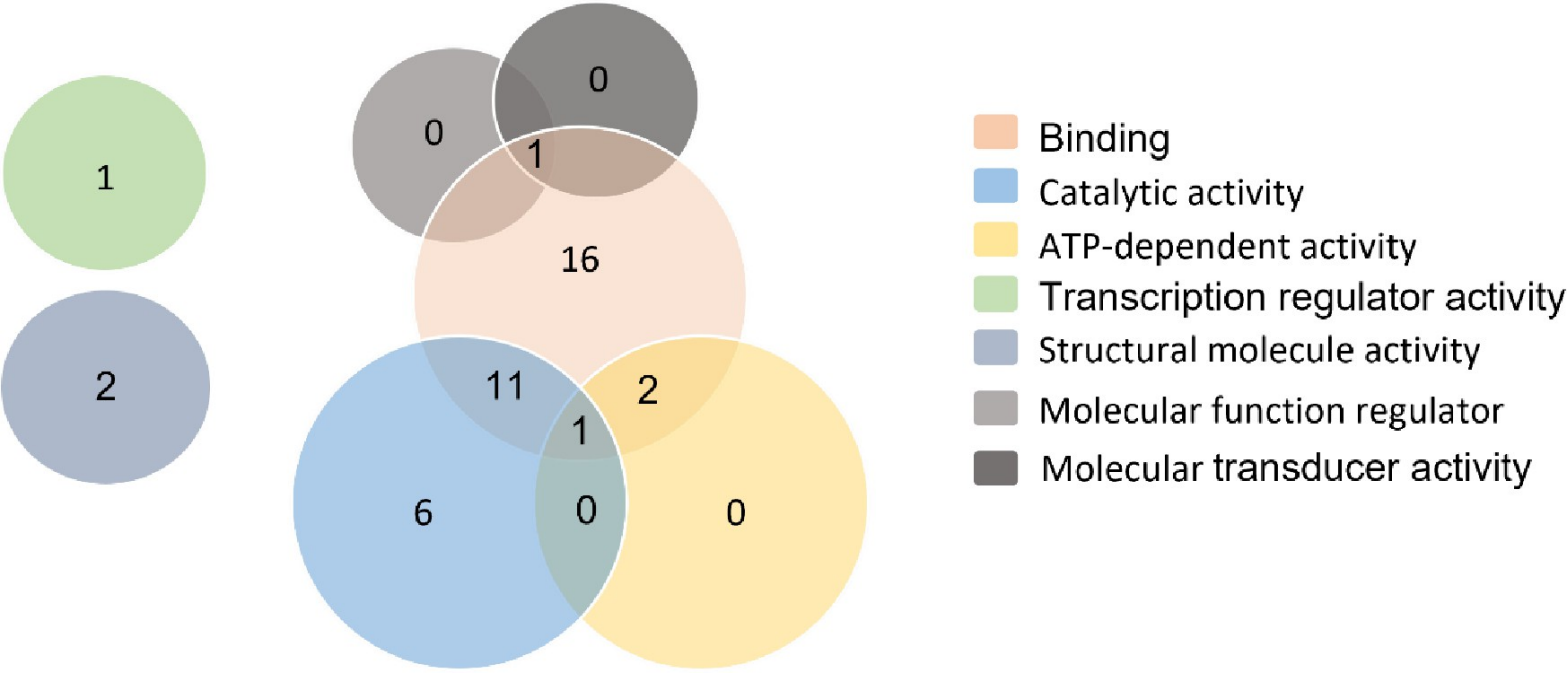
Gene ontology analysis. The numbers within the diagram represent the number of characterized sequences in each category.

The 40 analyzed in the molecular function category of GO exhibited 57 predicted functions. The identified proteins were primarily associated with binding (54.39%; n = 31) and catalytic activity (31.58%; n = 18). In addition to these main functions, there were proteins with ATP-dependent activity (n = 3), structural molecule activity (n = 2), molecular function regulation (n = 1), molecular transducer activity (n = 1) and transcriptional regulatory activity (n = 1) (S5 Table). Notably, several markers, including Lvan96, Lvan183, Lvan195, LvE22X, LvE26X, Livan04, CNM-MG-367, CNM-MG-369, CNM-MG-372, CNM-MG-390, CNM-MG-548, c2438/f35p18/1618, c16138/f14p37/618, CNM-MG-479, Lvan93, TUMXLv10.484, and TUMXLv9.174, were associated with more than one molecular function (Fig 2). On the other hand, 22 putative marker proteins did not have annotations for a molecular function (S5 Table).

### Prospecting of STR markers and population analysis by genotyping

The nine markers located in exons described in Table 3 were evaluated in a population to verify their potential for population studies and potential variations in each coding region.

**Table 3:** Markers used for in vitro validation.

Locus	Gene	Forward Primer	Reverse Primer	Repeat Motif	Reference
Lvan93	ATP-dependent Clp protease ATP-binding subunit clpX-like, mitochondrial	TCACCTATTCACTCTCAAAC	AGTGAGTGAGTTAGTGTGTTG	(AC)6	[74]
Lvan183	Sorbitol dehydrogenase-like	GTGAAGCCTCTCATTACTC	CATGACTACCAAGATTTCTC	(GA)7	[74]
Lvan195	26S proteasome regulatory subunit 6B-like	AAACTCGGTAGACTAATCC	TCCTCTCTCAAAAGTCAAG	(ATT)8	[74]
Lvan204	Serine protease inhibitor I/II-like	AGAACTGAACTTTGACCTTG	CATACAATTCCAAGACCG	(TTC)5	[74]
Livan04	Receptor-type tyrosine-protein phosphatase T-like	ATTCTTGGAGTATGCGGTGG	TGATTTGAGAACGAGACGGA	(ATTT)9	[3]
Livan44	Uncharacterized LOC113818723	ACCCTCTCATCAAGCAGTGG	TCCACAGAAGAGCGTGTTTG	(TC)8	[3]
Livan51	Inverted formin-2-like	CAATTACTCCGGCCTCAAGA	AACCGTACACAGGCCAATTC	(AG)8	[3]
CNM-MG-402	Prefoldin subunit 3-like	CTTTTGGCTGGCTTAC	TTCCTTTTGATCTACATTG	(AGAAA)3	[63]
CNM-MG-421	Eukaryotic peptide chain release factor subunit 1	TTTCTGCCACGGAGTT	CTGTTGCCCAAATAGC	(AAT)5	[63]

The average number of alleles per locus was approximately five, ranging from 1 to 8. However, the marker Livan44 did not exhibit any polymorphisms. Among the markers, Lvan93 displayed the highest heterozygosity (Ho = 1.000), while Livan04 had the lowest (Ho = 0). The markers Livan44, Lvan183, and Lvan93 showed significant departures from Hardy-Weinberg (HW) equilibrium based on the probability test. The markers CNM-MG-402 and CNM-MG-421 did not amplify in our population (Table 4). Although the primer Lvan204 could be amplified, population analysis was not conducted for this marker.

**Table 4:** Genetic parameters considering six microsatellite loci.

Locus	Repeat Motif	N° of animals tested	N° of alleles per locus	Size range	Ho	He	P-WHE	Fis (W&C)
Livan 04	(ATTT)9	15	4	140–170	0.857	0.564	0.247	-0.4928
Lvan 195	(ATT)8	15	5	90–110	0.444	0.753	0.403	0.4576
Livan 44	(TC)8	15	?	250–260	-	-	-	-
Livan 51	(AG)8	15	7	160–190	0.727	0.802	0.010	0.1398
Lvan183	(GA)7	50	5	230–290	0.789	0.679	0.000*	-0.1497
Lvan93	(AC)6	30	8	110–125	1.000	0.859	0.001*	-0.0980

Ho: observed heterozygosity; He: Expected heterozygosity; Fis: Endogamy indexing index; *not in Hardy-Weinberg equilibrium (p< 0.05)

The inbreeding coefficient (FIS) was less than zero for Livan04, Lvan183, and Lvan93, indicating higher genetic variability and the coexistence of different protein isoforms within the population (Table 4).

**Fig 3:**
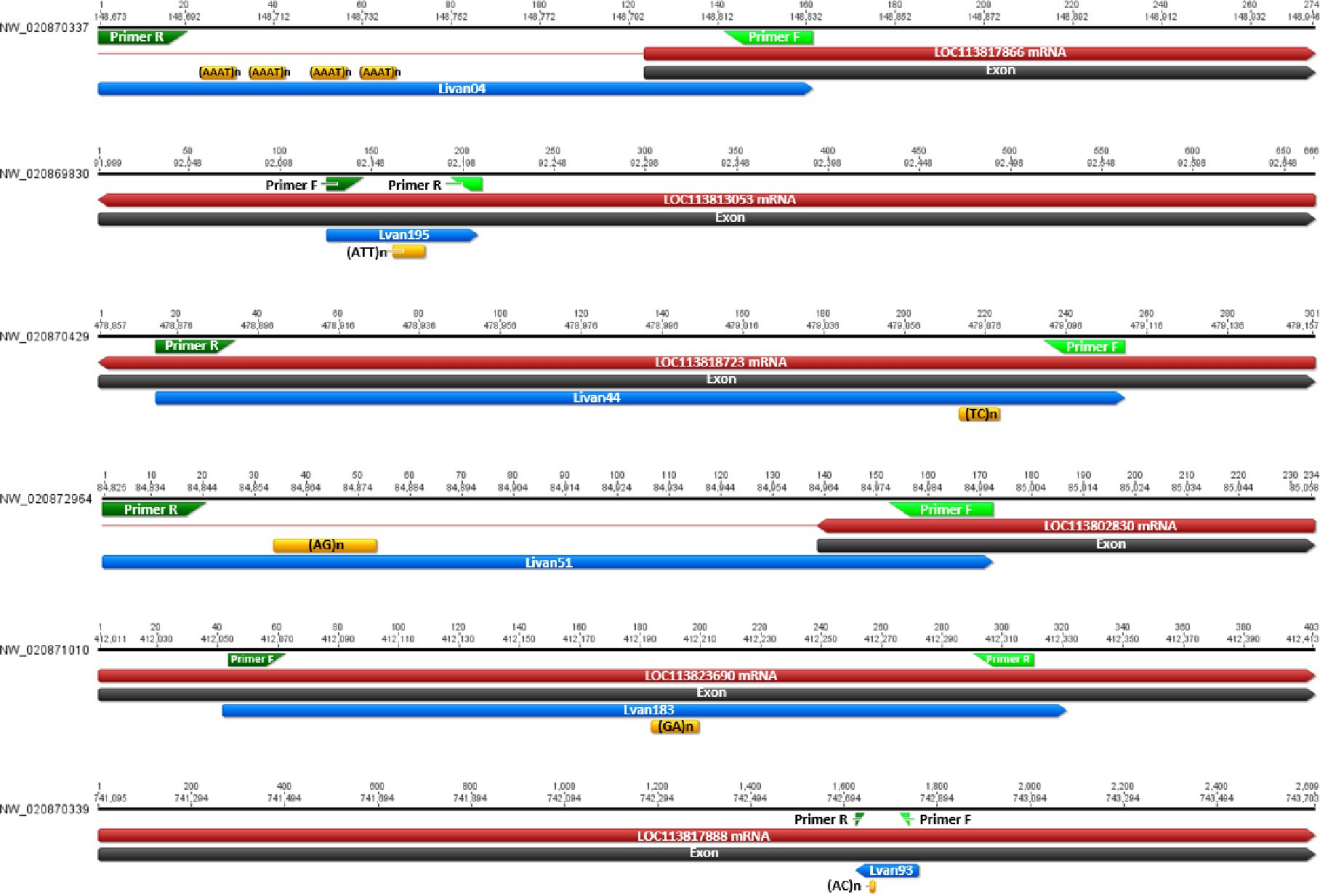
STRs located in exons within the *P*. *vannamei* genome. The marker sequences (shown in blue) represent the entire amplicon and include the primers (dark green and light green). The repeats within the markers are also indicated (orange). Exon annotations are presented in black.

The positions where these markers overlap in the exon are depicted in Fig 3. As they are situated within exon regions, variations in the number of their repetitions can alter the codons, thus potentially modifying the function of the protein. The majority of these STRs were located at the 3’ end of the mRNAs.

## Discussion

This study aimed to gather and consolidate all the currently available information on microsatellite markers characterized for *P*. *vannamei*. We believe that this represents a significant milestone, as data on these markers were previously scattered and, in many cases, lacked information regarding their genomic location. This was primarily since most of these markers were designed prior to the first genomic draft of *P*. *vannamei* [21]. The publication of the genomic draft showed the occurrence of 44 linkage groups, mapping to 44 distinct chromosomes. This allowed us to precisely map most of the markers to a linkage group, providing valuable information for breeding programs.

Overall, our observations revealed a high occurrence of diverse repeat motifs (ranging in length from 1 to 6 bp) within perfect short tandem repeats (STRs), as depicted in Fig 1. Among these, markers featuring dinucleotide repeats were the most prevalent, followed by trinucleotide and tetranucleotide repeats, which aligns with the findings reported in the original studies [21, 69, 72].

The main challenge encountered in this study was the task of establishing the linkage between these markers and their respective genomic locations. Up until now, no prior research has been conducted to connect the existing microsatellite markers (with available sequences) to their specific genomic positions. It was quite surprising to discover that numerous microsatellites in *P*. *vannamei* are situated within genomic regions that potentially bear some adaptive significance, as demonstrated in Table 2 and Fig 2. Functional annotation analysis indicated that out of the perfect markers, 62 (45.9%) were located within coding regions. In other words, these genes are associated with various processes such as host cell response to pathogens, energy metabolism, cytoskeleton organization/reorganization, protein folding, and the efficiency of translation mechanisms.

During our analysis, we observed redundancy and ambiguity in certain markers. Utilizing the Primer BLAST search, we found that the primers for the following five markers aligned with multiple locations in the genome. These locations could either be within the same genomic scaffolds or in different scaffolds, with the possibility of being on the same chromosome or not. The markers and their respective counts are as follows: CNM-MG-369 (n = 2), CNM-MG-444 (n = 3), CNM-MG-474 (n = 2), c7152/f1p0/6395 (n = 3), and c16138/f14p37/618 (n = 19). Further details can be found in S3 Table. This result confirms the existence of regions that are likely to arise from duplication events, a common feature in the *P*. *vannamei* genome also observed in other shrimp genomes [21]. Consequently, the use of these markers requires caution since they might be inaccurate when used in the traditional way.

Five short tandem repeats (STRs) were successfully validated in a shrimp population, confirming the occurrence of protein variants within the population, as outlined in Table 4. Some markers within this panel have demonstrated potential for application in traditional population studies. However, we believe that the true value of this panel lies in the correlation between protein variations and phenotypic traits. The observed deviations from the Hardy-Weinberg equilibrium in certain markers could potentially be explained by specific selective pressures acting on these protein isoforms.

The expansion or contraction of STRs can influence various biological processes, including gene transcription, splicing, and translation, thereby contributing to genomic plasticity [17]. The following are examples of proteins that exhibit different isoforms within the analyzed shrimp population, along with their respective functions:

1. Clp proteases are ATP-dependent enzymes that play a crucial role in the cleavage of proteins and polypeptides by hydrolyzing peptide bonds. They serve as precise regulatory mechanisms involved in protein degradation and quality control processes within cells [76, 77].
2. Sorbitol dehydrogenase (SDH) is an enzyme that relies on zinc as a cofactor and is primarily responsible for the conversion of D-sorbitol into D-fructose. This enzymatic activity holds significant importance in the early embryonic development of invertebrates [78]. In a study conducted by [79], rainbow trout were used to investigate the effects of increasing stock density on sorbitol dehydrogenase (SDH) activity. The findings revealed that higher stock densities in tanks had a significant inhibitory effect on SDH activity. Consequently, this led to the accumulation of sorbitol, disrupting the physiological balance of the fish, and resulting in undesirable outcomes. In *P*. *vannamei*, the expression levels of SDH have been found to be associated with Taura Syndrome Virus (TSV) infection.
3. The 26S proteasome regulatory is a large multi-protein complex that selectively degrades ubiquitin-tagged proteins, and this process is ATP-dependent [80–82]. It plays a key role in biological homeostasis since removing damaged or misfolded proteins [83, 84]. Furthermore, the ubiquitin-proteasome system may play an important role in crustaceans’ immune response and can be a candidate gene involved in host viral disease defense [85, 86]. [87] found a Ubiquitin proteasome mediated response in animals infected with WSSV to reduce the deleterious damage caused by the viral infection.
4. Protein tyrosine phosphatases (PTPs) are signaling molecules responsible for dephosphorylating tyrosine residues in proteins and play critical roles in cellular regulation and cell adhesion [88, 89]. In a study conducted by [89] using *P*. *vannamei*, it was discovered that the expression of non-receptor protein tyrosine phosphatase 6 (PTP6) was regulated by the interferon regulatory factor (IRF). Additionally, PTP6 was found to promote STAT dimerization, leading to increased expression of genes associated with STAT-targeted immune effectors. This, in turn, enhanced the antiviral immunity of shrimp.
5. Inverted Formin plays a diverse range of roles in actin polarization and depolarization processes. It is involved in various cellular processes, including cytokinesis, cell adhesion, cell motility, endocytosis, among others. Additionally, studies have reported its interaction with microtubules, which contributes to microtubule stabilization [90].

## Conclusion

This study provides an updated characterization of previously validated microsatellite markers in population studies for *P*. *vannamei*. Among the 306 markers analyzed, 117 were identified as perfect repeats/markers, with 62 located within coding regions of the genome. Functional characterization revealed their potential influence on cellular processes, including binding function, catalytic activity, and transcriptional activity, which may be linked to phenotypic traits. Additionally, a panel of five markers located in coding regions was validated in vitro, confirming the presence of different protein isoforms within the *P*. *vannamei* population. The results highlight the potential of coding region markers in establishing direct associations between phenotype and genotype. The validation of six markers in vivo further supports this notion. Moreover, the study suggests the possibility of exploring the remaining untested markers through in silico functional predictions, which would expand the potential for phenotype-genotype associations.

## Supporting information

S1 Table List of *Penaeus vannamei* microsatellite markers. (XLSX)

S2 Table Perfect *Penaeus vannamei* microsatellite markers that have been validated in population studies. (XLSX)

S3 Table Coding sequences in which the microsatellites are located. (XLSX)

S4 Table Gene predictions obtained from the identified sequences after functional annotation. (XLSX)

S5 Table Results of the gene ontology analysis. (XLSX)
